# Inside-Outside Mastoidectomy for Middle Ear Paragangliomas: Surgical Technique and Outcomes

**DOI:** 10.7759/cureus.97180

**Published:** 2025-11-18

**Authors:** Carlos Ríos-Deidán, Mercedes Narvaez, Diana Salgado Guarderas, Diego Guaquipana, Tamara Acosta

**Affiliations:** 1 Facultad de Ciencias Médicas, Universidad Central del Ecuador, Quito, ECU; 2 Otorhinolaryngology Unit, Hospital de Especialidades Carlos Andrade Marín, Quito, ECU; 3 Otorhinolaryngology Department of Medical Sciences Faculty, Pontifical Catholic University of Ecuador, Quito, ECU; 4 Otorhinolaryngology Unit, Hospital DAME-Hospital de Especialidades Médicas en Quito, Quito, ECU

**Keywords:** glomus tympanicum, inside-outside mastoidectomy, skull base, surgical approach, tumor

## Abstract

Background: Middle ear paragangliomas (glomus tympanicum) are rare vascular tumors that pose significant surgical challenges due to their location and vascularity. The inside-outside mastoidectomy combines transcanal and retroauricular approaches to optimize resection and preserve hearing.

Methods: We conducted a retrospective observational study of eight female patients with stage A1-B3 glomus tympanicum treated at Carlos Andrade Marín Specialty Hospital between 2018 and 2024. Clinical presentation, imaging, intraoperative findings, complications, and audiological outcomes were analyzed.

Results: All patients presented with conductive hearing loss and pulsatile tinnitus. Complete tumor resection was achieved in 100% of cases with mean intraoperative bleeding of 200 ± 82 milliliters and mean follow-up of 38 ± 18 months. Complications included three tympanic membrane perforations (37.5%) and one transient facial palsy (12.5%). Hearing improvement was documented in 37.5% of patients. No tumor recurrences were observed during follow-up.

Conclusion: Inside-outside mastoidectomy is a safe and effective approach for the management of middle ear paragangliomas, offering excellent tumor control and favorable hearing outcomes. Further studies with larger cohorts are warranted to validate these findings.

## Introduction

Glomus tympanicum, also referred to as middle ear paragangliomas, is a rare vascular tumor arising from paraganglionic tissue derived from the embryonic neural crest. These tumors are slow-growing, locally aggressive, and located in close proximity to critical neurovascular structures such as the jugular bulb, internal carotid artery, facial nerve, and cranial nerves IX-XII. Although generally benign, approximately 10% are multicentric, and another 10% show familial inheritance [1-3].

The most common presenting symptoms are conductive hearing loss and pulsatile tinnitus [4]. Imaging studies such as contrast-enhanced computed tomography (CT), magnetic resonance imaging (MRI), and magnetic resonance angiography (MRA) are essential for diagnosis and staging [5]. Surgical excision is the mainstay of treatment, but the procedure is technically challenging due to the tumor’s vascularity and location, which can endanger the ossicular chain, facial nerve, and carotid artery [5-7].

The Fisch-Mattox classification is widely used to stage these tumors [5-8]. In 1977, Fisch introduced the infratemporal fossa approach, which standardized surgical management of skull base paragangliomas.

In this study, we present our experience with a modified “inside-outside” mastoidectomy, which combines a transcanal and posterior approach to optimize tumor removal, visualization, and hearing preservation.

Objectives

To evaluate the effectiveness of the inside-outside mastoidectomy in achieving complete tumor resection of stage A1-B1 glomus tympanicum. To assess postoperative hearing outcomes and complication rates associated with this technique. To conceptually compare these results with those reported for traditional microscopic and endoscopic approaches in the literature.

## Materials and methods

Study design and setting

We conducted a retrospective observational study at the Otorhinolaryngology Unit of Carlos Andrade Marín Specialty Hospital (HECAM, Quito, Ecuador). Clinical data were obtained from the institution’s electronic medical record system and operative reports, reviewed retrospectively. The study was approved by the institutional review board (Approval No. 004, April 16, 2020).

Patients

We included all patients diagnosed with glomus tympanicum (Modified Fisch-Mattox stages A1-B1) who underwent inside-outside mastoidectomy between January 2018 and December 2024. Patients with incomplete records, previous surgery for temporal bone paraganglioma, or familial/multicentric disease were excluded.

Preoperative workup

All patients underwent contrast-enhanced temporal bone computed tomography (CT) for diagnosis and staging. Magnetic resonance imaging (MRI) with angiographic sequences (MRA) was performed in six of eight patients (75%) to assess tumor extent and vascular relationships and to rule out jugular paraganglioma. No preoperative embolization was required. Screening for multicentric or familial disease included clinical examination, neck and abdominal ultrasound, and detailed family history. None of the patients demonstrated synchronous lesions or hereditary patterns.

Operative setup and surgical team

All procedures were performed at the tertiary otologic surgery unit of Carlos Andrade Marín Specialty Hospital, equipped with high-definition surgical microscopes and 0° and 30° rigid endoscopes. Surgeries were carried out under general anesthesia by the same senior otologic surgeon. The operative field was prepared with bipolar cautery, suction irrigation, and otologic microinstruments.

All procedures were performed under general anesthesia with the patient in the supine position and the head rotated contralaterally. A standardized inside-out mastoidectomy was carried out in all cases (tumor stages A1-B1) through a retroauricular approach, using combined microscopic and endoscopic visualization.

After skin incision and elevation of the tympanomeatal flap, the middle ear and tumor margins were exposed. Under microscopic guidance, bone drilling was initiated from the middle ear toward the mastoid cavity (inside-out technique), progressively unroofing the epitympanum and hypotympanum to delineate the lesion and its extensions. This dissection vector provided direct visualization of the mesotympanum and facial recess while preserving the posterior canal wall. Key anatomical landmarks included the promontory, facial nerve tympanic segment, chorda tympani, and ossicular chain.

Endoscopic assistance with 0° and 30° rigid scopes enhanced visualization of hidden recesses, particularly the epitympanum, sinus tympani, and hypotympanum, allowing precise removal of residual tumor tissue and confirmation of complete clearance. The combined optical control minimized blind areas and reduced manipulation of the ossicular chain.

After full tumor excision, tympanoplasty was performed, using cartilage or temporalis fascia grafts as indicated. Hemostasis was achieved by bipolar cautery and cotton pledgets soaked in 1:1000 epinephrine solution, applied intermittently for 30-60 seconds. Final endoscopic inspection through the external auditory canal confirmed complete resection before wound closure.

All procedures were performed by a single senior otologic surgeon using standard otologic instrumentation and a uniform intraoperative protocol.

**Figure 1:**
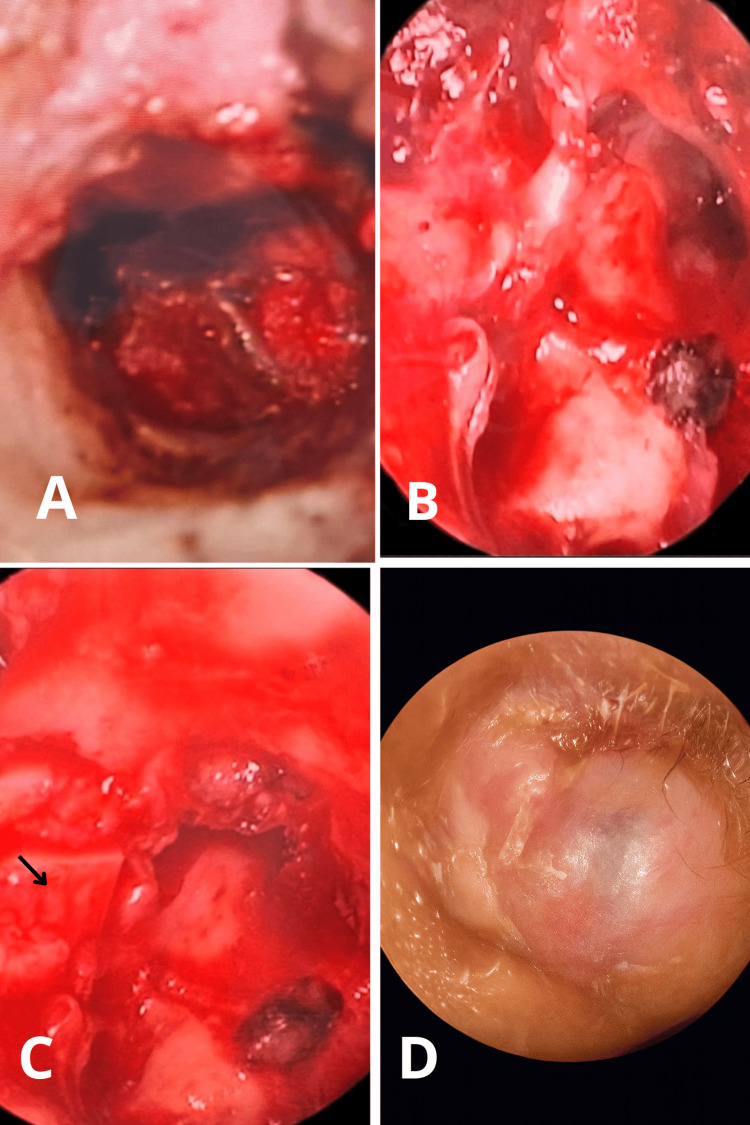
Intraoperative findings A: Microscopic findings of glomus tumor. B: Surgical resection of a glomus tumor. C: Attic exclusion with cartilage. D: One-year postoperative outcome.

Intraoperative visualization confirmed tumor location, and hemostasis was achieved (Figure 1).

Follow-up protocol

Postoperative assessment included otoendoscopy at three months, followed by annual controls. Contrast-enhanced CT was performed at three months to exclude residual disease. Audiometric evaluation was conducted preoperatively and at the last follow-up.

Outcomes measured

Hearing outcomes were assessed by comparing pre- and postoperative pure-tone averages (PTA) at 0.5, 1, 2, and 4 kHz. Hearing improvement was defined as a ≥10 dB PTA reduction or ≥10 dB air-bone gap closure; preservation was defined as a change within ±10 dB; deterioration was defined as ≥10 dB worsening in PTA. Complications and recurrence were recorded at each follow-up visit.

Statistical analysis

Data were analyzed using descriptive statistics. Continuous variables were reported as mean ± standard deviation, and categorical variables as absolute frequencies and percentages.

## Results

**Figure 2:**
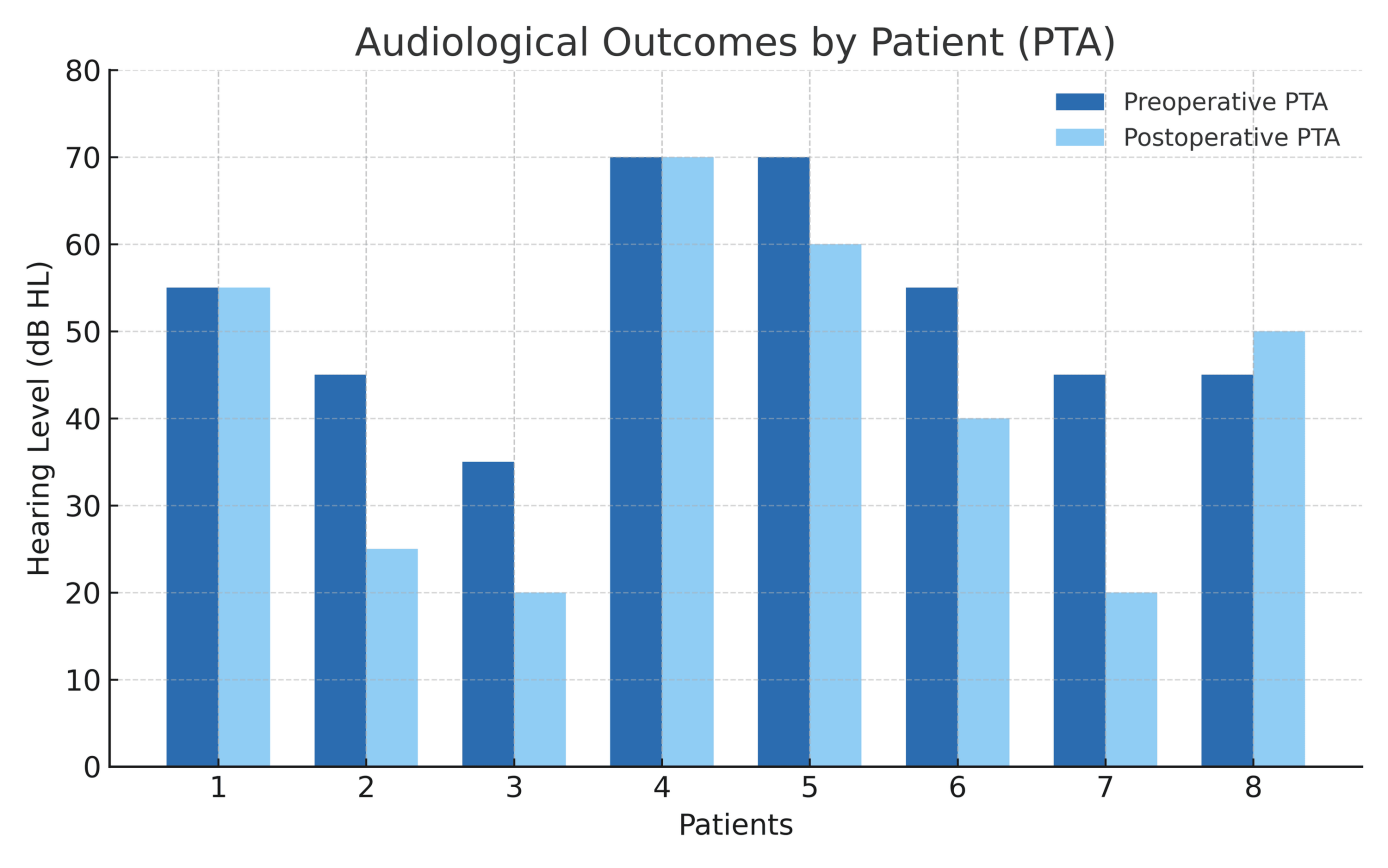
Audiological characteristics (hearing average)

Eight female patients (mean age, 57.8 ± 13 years) underwent an inside-out mastoidectomy for middle ear paraganglioma. The most frequent presenting symptoms were conductive hearing loss and pulsatile tinnitus (Table 1). On otoendoscopy, all cases showed a reddish trans-tympanic vascular mass consistent with Brown’s sign (Figure 2).

**Table 1:** Clinical and surgical characteristics The tympanic perforation was repaired with cartilage after a year. MFM: Modified Fisch-Mattox Classification; GY: Jugular Bulb; M: Months; mm: Millimeter Source: Database (Carlos Andrade Marín Hospital).

Case	Age	Side	MFM	Tumor Size (mm)	Follow-up in months/recurrence	Post-operative Imaging
1	69	Left	A2	10	60M/NO	12M/YES
2	49	Left	A2	12	60M/NO	12M/YES
3	33	Right	A2	7	52M/NO	12M/YES
4	66	Left	B1	13	30M/NO	12M/YES
5	57	Right	B3	20	24M/NO	12M/YES
6	68	Right	B3	11	36M/NO	12M/YES
7	63	Left	A2	8	20M/NO	12M/YES
8	29	Right	B2	8	6M / NO	6M/YES

Average intraoperative blood loss was 200 ± 82 mL, and the mean follow-up period was 38.8 ± 17.7 months. Two patients required conversion to a more extensive procedure because intraoperative findings revealed B3-stage extension.

Postoperative complications were limited to three tympanic membrane perforations (37.5%) and one transient facial palsy (12.5%). The residual perforations were successfully repaired with cartilage grafts about eight to twelve months after the initial surgery, and all healed uneventfully without further morbidity or hearing decline.

Three patients (37.5%) experienced objective hearing improvement after surgery. No recurrences were identified during the observation period.

When hearing results were analyzed according to tumor stage, patients with A1-A2 lesions typically had mild-to-moderate conductive loss preoperatively and demonstrated greater postoperative gains, while those with B2-B3 tumors had more advanced hearing loss and limited recovery potential.

Overall, three of eight patients (37.5%) showed significant improvement, with an average PTA gain of 12 ± 5 dB. Hearing thresholds remained stable (±10 dB) in the remaining patients. No tumor recurrence was observed during follow-up (mean 38 ± 18 months; minimum, six months).

## Discussion

Glomus tympanicum tumors account for approximately 70% of temporal bone paragangliomas and show a marked female predominance with peak incidence between the fourth and sixth decades of life. Our findings are consistent with these epidemiological patterns, as all patients in our cohort were female with a mean age of 57.8 years.

Surgical management of glomus tympanicum remains challenging due to the tumor’s vascularity and its proximity to critical structures such as the ossicular chain, carotid artery, and facial nerve. For small A1 tumors, purely transcanal approaches, either microscopic or endoscopic, are often sufficient. However, larger or more advanced lesions typically require combined or more extensive approaches.

The inside-outside mastoidectomy described in our series integrates the advantages of both transcanal and retroauricular access. This allows for improved visualization of the middle ear cleft and safer dissection around vital neurovascular structures. In our cohort, complete tumor resection was achieved in 100% of cases, with no recurrences during a mean follow-up of over three years. These results are comparable or superior to outcomes reported with purely endoscopic or modified mastoidectomy techniques [9,10,11-15].

Notably, hearing improvement was achieved in 37.5% of patients, and stable hearing was preserved in the remainder. Complication rates were acceptable, limited to tympanic membrane perforations and a single transient facial palsy, all of which resolved without long-term morbidity. These findings suggest that the inside-outside approach not only ensures tumor control but also maximizes functional preservation, particularly of hearing.

Our institutional experience supports previously published reports highlighting the utility of combined approaches in early- to mid-stage paragangliomas [9,10,12-17]. By adapting the extent of mastoidectomy to intraoperative findings and tumor stage, we were able to optimize both oncologic and functional outcomes.

To illustrate these adaptations, Table 2 compares the surgical algorithms proposed by Yilala and Sanna with the modifications applied at our institution. This comparison highlights how the inside-outside mastoidectomy was tailored to tumor stage while preserving oncologic safety and functional outcomes (Table 2).

**Table 2:** Comparative table of surgical algorithms: Yilala & Sanna classification vs. surgical approach at HECAM Source: Yilala MH, Fancello G, Fancello V, Lauda L, Sanna M. Long-Term Surgical Outcome of Class A and B Tympanomastoid Paragangliomas. Cancers (Basel). 2024 Apr 11;16(8):1466. [10,17]

Classification	Stage	Surgical Approach	Adaptation for Carlos Andrade Hospital
A	A1	Transcanal approach	Transcanal – retroauricular approach
	A2	Transcanal – retroauricular approach	Mastoidectomy Inside-Out
B	B1	Canal wall up mastoidectomy with posterior tympanotomy	Mastoidectomy Inside -out
	B2	Canal wall mastoidectomy	Canal Wall down Mastoidectomy
	B3	Posterior tympanotomy	Canal Wall down Mastoidectomy

The main limitation of this study is the relatively small sample size and its retrospective design, which precludes direct statistical comparison with alternative approaches. Nevertheless, it represents one of the first Latin American institutional series applying this combined technique, and it provides valuable data for centers facing similar surgical challenges. Future multicenter studies with larger cohorts are needed to confirm the reproducibility and long-term advantages of this approach.

## Conclusions

The inside-outside mastoidectomy achieved complete tumor resection and preserved or improved hearing in most patients, with no recurrences during the available follow-up. By combining transcanal and retroauricular access, this approach appears to be safe and effective within the limitations of this retrospective series, offering a practical alternative for early- to mid-stage glomus tympanicum. Comparisons with purely endoscopic or microscopic techniques are literature-based and should be interpreted conceptually rather than empirically.

Although no recurrences were observed during follow-up, this finding should be interpreted cautiously, as one patient had a short postoperative surveillance period of only six months. A longer follow-up is required to confirm sustained tumor control.
